# Expanded Normal Weight Obesity and Insulin Resistance in US Adults of the National Health and Nutrition Examination Survey

**DOI:** 10.1155/2017/9502643

**Published:** 2017-07-25

**Authors:** Keilah E. Martinez, Larry A. Tucker, Bruce W. Bailey, James D. LeCheminant

**Affiliations:** Department of Exercise Sciences, College of Life Sciences, Brigham Young University, Provo, UT 84602, USA

## Abstract

This study aims to expand the evaluation of normal weight obesity (NWO) and its association with insulin resistance using an NHANES (1999–2006) sample of US adults. A cross-sectional study including 5983 men and women (50.8%) was conducted. Body fat percentage (BF%) was assessed using dual-energy X-ray absorptiometry. Expanded normal weight obesity (eNWO) categories, pairings of BMI and body fat percentage classifications, were created using standard cut-points for BMI and sex-specific median for BF%. Homeostatic model assessment-insulin resistance (HOMA-IR) levels were used to index insulin resistance. Mean ± SE values were BMI: 27.9 ± 0.2 (women) and 27.8 ± 0.1 (men); body fat percentage: 40.5 ± 0.2 (women) and 27.8 ± 0.2 (men); and HOMA-IR: 2.04 ± 0.05 (women) and 2.47 ± 0.09 (men). HOMA-IR differed systematically and in a dose-response fashion across all levels of the eNWO categories (*F* = 291.3, *P* < 0.0001). As BMI levels increased, HOMA-IR increased significantly, and within each BMI category, higher levels of body fat were associated with higher levels of HOMA-IR. Both high BMI and high BF% were strongly related to insulin resistance. Insulin resistance appears to increase incrementally according to BMI levels primarily and body fat levels secondarily. Including a precise measure of body fat with BMI adds little to the utility of BMI in the prediction of insulin resistance.

## 1. Introduction

Over the past several decades, the number of adults in America with excess body weight has increased substantially [1]. According to body mass index (BMI) data derived from the National Health and Nutrition Examination Survey (NHANES), approximately 44% of adults were overweight or obese in 1976–1980 [2]. Findings for 2011-2012 indicate that the prevalence increased to 69% of adults [3], an increase of over 55%. This rising trend is not without serious consequences.

Obesity is a significant risk factor for numerous medical conditions, including insulin resistance and metabolic disease. In a recent paper, Lim et al. showed that obesity, whether measured by BMI or body fat percentage, is highly correlated with insulin resistance, as indexed by elevated homeostatic model assessment (HOMA-IR) levels [4]. Additionally, BMI is a serious risk factor for the development of type 2 diabetes, displaying a dose-response relationship [5]. Research also shows that adults who have normal body weight, but excess body fat, are at increased risk for developing metabolic syndrome and insulin resistance [6].

Though frequently used to classify obesity, BMI does not measure adiposity. BMI is calculated using only height and weight, not body composition [7]. BMI often misclassifies those with excess adiposity (high body fat percentage) as normal or healthy [8]. Though BMI has high specificity for predicting high body fat percentage [9, 10], several researchers have found that BMI has low sensitivity for predicting body fat percentage [9–11].

Many individuals assume that because they have a normal body weight, they are metabolically healthy, and those who are overweight may assume that they are metabolically unhealthy. Though commonly used, body weight, and particularly BMI, is not a high-quality index of health status.

Researchers have tried to remedy the problems associated with using BMI to index overweight and obesity. As a result, the concept of normal weight obesity (NWO) has emerged. NWO, a condition in which individuals are classified as normal weight by BMI, but have excess body fat (defined differently by various researchers), has not been researched extensively, but it seems to be a good predictor of multiple health risks. In recent studies, NWO has been associated with metabolic dysregulation [12], physical impairment [13], and cardiovascular mortality [14].

NWO is a good predictor of insulin resistance. Research by Romero-Corral et al. shows that adults with NWO have four times higher prevalence of metabolic syndrome compared to their counterparts and that insulin sensitivity tends to decrease as body fat percentage increases [15]. Other researchers have also shown that body fat percentage predicts insulin resistance [16, 17]. Research by Madeira et al. indicates that those with NWO have six times greater risk for metabolic syndrome than those without NWO [6]. Additionally, adults with sarcopenic obesity [18], low muscle mass, and elevated body fat also tend to have higher levels of metabolic syndrome [19], insulin resistance [20, 21], and several cardiovascular risk factors [20], compared to their counterparts.

Although promising, NWO is a limited index, including just one BMI category (normal weight) and one body fat category (high body fat). There are many other possible BMI and body fat combinations, such as underweight-low body fat (UW-L), underweight-high body fat (UW-H), normal weight-low body fat (NW-L), normal weight-high body fat (NW-H), overweight-low body fat (OW-L), overweight-high body fat (OW-H), obese-low body fat (OB-L), and obese-high body fat (OB-H). In the present study, these categories are referred to as expanded normal weight obesity (eNWO). To date, few of many studies have investigated multiple BMI and body fat category pairings and their relationship with metabolic dysregulation. Moreover, when researchers have studied some BMI and body fat pairs, they have used bioelectrical impedance [14, 19, 15] or skinfolds [6] to estimate body fat, as opposed to more precise and reliable measurement tools.

The purpose of this investigation was to expand the evaluation of NWO and its association with insulin resistance using a nationally representative sample of US adults. A secondary purpose was to overcome weaknesses of other obesity and metabolic dysregulation research, particularly to assess body fat percentage using a high-quality measurement method, dual energy X-ray absorptiometry (DXA), and to control for potentially confounding factors, such as age, sex, race, smoking, and physical activity.

## 2. Methods

### 2.1. Design

A cross-sectional study was performed to examine the relationship between an expanded version of NWO (eNWO) and insulin resistance in US adults. Data on eNWO and insulin resistance were obtained using BMI, body fat percentage, and HOMA-IR from the National Health and Nutrition and Examination Surveys (1999–2006). NHANES is an extensive stratified data set representative of the noninstitutionalized civilian population of the United States [22].

### 2.2. Participants

Subjects 20–84 years old with information on age, sex, race/ethnicity, BMI, body fat percentage, fasting blood glucose and insulin levels, physical activity, and smoking status were included in the analyses. The number of subjects who had both fasting insulin and fasting glucose data (used to calculate HOMA-IR) was 12,561. Including only adults 20–84 years old resulted in 8331 subjects. Narrowing the sample to those without diabetes and adults not taking medication for diabetes, the sample was 7249. Some subjects had missing data for the exposure variable or one of the covariates, resulting in a total of 5983 subjects. All measurement procedures were taken from the published guidelines and procedures used by the National Health and Nutrition Examination Survey [23, 24].

### 2.3. Instrumentation and Measurements

The criterion variable of the current investigation was insulin resistance, indexed using the homeostatic model assessment (HOMA-IR). The primary predictors were body mass index (BMI) and body fat percentage. Together, these measures were used to create a new variable called expanded normal weight obesity (eNWO).

#### 2.3.1. Weight and Height

Weight was taken using a Toledo digital scale while the subject was wearing only underwear, a disposable paper gown, and foam slippers [24, 25]. Standing height was measured with a fixed stadiometer with a moveable headboard [25].

#### 2.3.2. Body Mass Index (BMI)

BMI was calculated using the formula: weight (kg) divided by height in meters squared [26]. Standard cut-points were used. Underweight was defined as a BMI < 18.5, normal weight included BMI values between 18.5 and <25.0, overweight included BMI values from ≥25.0 to <30.0, and obesity was defined as a BMI of >30.0 kg/m^2^ [26].

#### 2.3.3. Body Composition

Body fat percentage using DXA was a measured variable in the NHANES 1999–2006 surveys only. Pregnant females were not scanned. The whole body DXA scan used a Hologic QDR 4500A fan-beam densitometer (Hologic Inc., Bedford, Massachusetts) [24, 25].

Participants taller than 6′5″ or more than 300 lbs were excluded due to DXA table limitations [24, 25]. Due to these exclusions for large body sizes, missing and invalid DXA data were not missing at random, leading the NHANES analysts to perform multiple imputations to complete the missing data for analysis. Details of the multiple imputation protocol are described elsewhere [24, 27].

#### 2.3.4. Insulin Resistance

Insulin resistance was indexed using HOMA-IR (fasting insulin (*μ*U/mL) × fasting glucose (mg/dL)/405). Fasting insulin and fasting glucose data were obtained through the NHANES measurements of diabetes profiles [28]. Adults with diabetes (defined as having a fasting blood glucose of ≥126 mg/dL, being told by a physician that one is diabetic, or using insulin or an oral medication for diabetes) were not included in the study.

Subjects assigned to NHANES morning sessions were asked to fast for 9 hours. Before blood collection, the phlebotomist administered a fasting questionnaire [28]. Detailed information is provided by NHANES about the insulin and glucose measurement protocols [28].

#### 2.3.5. Expanded Normal Weight Obesity (eNWO)

To study the relationship between eNWO and insulin resistance, pairings of BMI and body fat percentage were formed. Specific eNWO categories are defined in Table 1.

#### 2.3.6. Covariates

The study controlled for differences in age, sex, race, year of assessment, cigarette smoking, and physical activity. NHANES used the following race/ethnicity categories: non-Hispanic White, non-Hispanic Black, Mexican American, other races, including multiracial, and other Hispanics.

Cigarette smoking was indexed using pack-years. The number of cigarettes smoked per day and the number of years the person has smoked were multiplied and then divided by 20, resulting in a continuous variable, pack-years [29].

NHANES assessed participation in moderate and vigorous physical activity by employing two separate questions [29]. Moderate activity was assessed by asking subjects if they participated in moderate physical activity for at least 10 minutes over the last 30 days. Moderate activity was described as causing only light sweating or a slight to moderate increase in breathing or heart rate, as used in other research [30, 31]. Examples included brisk walking, bicycling for pleasure, golf, and dancing. Involvement in vigorous activity was measured similarly but was described as causing heavy sweating or large increases in breathing or heart rate [30]. Examples included running, lap swimming, aerobics classes, or fast bicycling.

### 2.4. Statistical Analysis

Results derived from NHANES research are special and unique because they can be generalized to the US noninstitutionalized, civilian population. This is because of random sampling and the use of person-level sample weights. When unequal selection probability is applied, the sample weights produce an unbiased national estimate.

In the present study, descriptive data, including frequencies for categorical variables and means ± standard errors for continuous variables, were reported. Each descriptive value included adjustments based on the sophisticated sampling design of NHANES by incorporating strata and primary sampling unit (PSU) indicators, as well as sample weights for the subsample of fasting participants used in the current study. Proc SurveyMeans was employed to generate weighted means that represent values for the US population, and Proc SurveyFreq was used to calculate weighted frequencies, which are also generalizable to the US adult population [32].

The primary outcome variable of the current study was insulin resistance, indexed using HOMA-IR. Because HOMA-IR distributions deviated significantly from normality, HOMA-IR values were transformed by natural logarithm prior to modeling.

For the current study, the exposure variable was normal weight obesity expanded to include all categories of BMI (eNWO), which has not been investigated previously. Typical BMI categories (underweight, normal weight, overweight, and obese) were used along with two categories based on body fat percentage (low body fat and high body fat). eNWO was a categorical variable reflecting each possible pairing between the four BMI categories and the two categories of body fat. In total, there were eight eNWO categories, as shown in Table 1. Both underweight categories were combined into one general underweight category because the number of subjects in each subgroup was low, leaving seven final eNWO categories.

The primary analysis was to determine the extent to which mean HOMA-IR values differed across the categories of eNWO using linear regression and the Proc SurveyReg procedure. Estimates for each regression model were based on the probability sampling strategy of NHANES. To test the hypothesis that the association between eNWO and insulin resistance is partially mediated by the covariates, these factors were controlled statistically using partial correlation. Adjusted means were calculated using the least-squares means procedure.

Secondary analyses were conducted to determine the extent to which HOMA-IR values differed across sex-specific BMI quintiles and also sex-specific body fat percentage quintiles. Again, partial correlation was employed to determine the influence of the covariates and the least-squares means procedure was used to provide adjusted means.

All *P* values were two-sided, and statistical significance was accepted when alpha was <0.05. The statistical analyses were computed using SAS Version 9.4 (SAS Institute Inc., Cary, NC).

## 3. Results

Descriptive information about the sample of 5983 participants is displayed in Table 2. Overall, nearly two-thirds of the sample was overweight or obese, according to standard BMI cut-points.

Mean BMI (±SE) was 27.9 ± 0.2 kg/m^2^ for women and 27.8 ± 0.1 kg/m^2^ for men, whereas average body fat percentage was 40.5 ± 0.2 and 27.8 ± 0.2 for women and men, respectively. Mean HOMA-IR was 2.04 ± 0.05 for women and 2.47 ± 0.09 for men.

As shown in Table 3, HOMA-IR levels differed systematically and in a dose-response fashion across all 7 of the eNWO categories (*F* = 291.3, *P* < 0.0001). As BMI levels increased, HOMA-IR increased significantly, and within each BMI category, higher levels of body fat were associated significantly with higher levels of HOMA-IR. Adjusting for differences in the demographic covariates, specifically age, sex, race, and year of assessment, modified the relationship between eNWO and HOMA-IR slightly (*F* = 286.2, *P* < 0.0001). However, controlling for differences in the lifestyle covariates, in addition to the demographic variables, strengthened the association between eNWO and HOMA-IR (*F* = 340.9, *P* < 0.0001).

HOMA-IR differences between the high and low body fat categories within each BMI level were meaningful. For example, within the normal weight BMI category, the HOMA-IR mean for the high body fat group was approximately 45% higher than it was for those in the low body fat category.

With subjects divided into BMI categories based on sex-specific quintiles, not standard BMI cut-points (Table 4), or body fat percentage sex-specific quintiles (Table 5), HOMA-IR differed significantly across the BMI groups (BMI5) (*F* = 448.5, *P* < 0.0001) and the body fat quintiles (BF%5) (*F* = 451.6, *P* < 0.0001), with no variables controlled. Adjusting for differences in all of the demographic and lifestyle covariates resulted in stronger associations between HOMA-IR and BMI5 (*F* = 464.4, *P* < 0.0001) and BF%5 (*F* = 511.3, *P* < 0.0001), as shown in Tables 4 and 5, respectively. The relationship between BMI5 and HOMA-IR was weakened substantially when all of the covariates and also BF%5 were controlled statistically (*F* = 141.9, *P* < 0.0001), as shown in Table 4. However, the association between BF%5 and HOMA-IR was weakened to a greater extent after adjusting for all of the covariates and also BMI5 (*F* = 68.8, *P* < 0.0001), as shown in Table 5.

The relationship between BMI and body fat percentage was moderate (*R*^2^ = 0.325, *F* = 2194.4, *P* < 0.0001) with both measures treated as continuous variables. With BMI and body fat percentage divided into sex-specific quintiles, the association was also significant (Wald chi-square = 1411.0, *F* = 88.2, *P* < 0.0001). Agreement among quintiles was modest. There was 67% agreement between quintile 1 for BMI5 and quintile 1 for BF%5. For quintile 5, agreement was similar (66%). However, for quintile 2, agreement was 37%, for quintile 3, agreement was 33%, and for quintile 4, agreement was 38%.

## 4. Discussion

According to the results, there appears to be an undeviating dose-response relationship for insulin resistance across each of the seven eNWO categories. Specifically, with each BMI category divided into low and high body fat groups, HOMA-IR varies according to body fat levels within each BMI category. This pattern remains consistent across the entire eNWO spectrum, without exception, and each HOMA-IR mean differs significantly from each other mean across every eNWO category (Table 3).

If body fat plays a more important role than BMI in insulin resistance, as suggested by Gomez-Ambrosi et al. [33], one would expect individuals with low body fat to have lower HOMA-IR levels than those in a neighboring lower BMI category with high body fat. For example, if body fat was key, then logic would suggest that participants in the overweight-low body fat category would tend to have significantly lower HOMA-IR levels than those in the normal weight-high body fat category. However, this was not supported by the present study. Instead, HOMA-IR moved incrementally according to BMI levels primarily and body fat levels secondarily. Specifically, individuals in the overweight-low body fat category had significantly higher HOMA-IR levels than those in the normal weight-high body fat category, and this pattern persisted across all of the eNWO categories.

Testing for differences in HOMA-IR across the sex-specific BMI (Table 4) and body fat percentage (Table 5) quintiles separately showed that the two body composition indexes have similar independent associations with HOMA-IR. However, after adjusting for differences in the covariates and BMI5 (BMI quintile), the relationship between body fat percentage quintile (BF%5) and HOMA-IR was attenuated more than when the covariates and BF%5 were controlled and the association between BMI5 and HOMA-IR was tested. Apparently, the relationship between BMI and HOMA-IR is much stronger after adjusting for body fat differences than the association between body fat and HOMA-IR, with BMI controlled.

Although both BMI and body fat percentage appear to play important roles in insulin resistance, NHANES data suggests that the contribution of BMI is greater than the contribution of body fat percentage. Therefore, given the costs of time, training, and equipment associated with measuring body fat to supplement BMI, and also the fact that BMI results remain the same whether or not body fat findings are included, for predicting HOMA-IR, the better choice may be to differentiate among adults based on BMI and not include a measure of body fat percentage.

To date, a limited number of studies have investigated the relationship between NWO and insulin resistance. However, few have used a reliable measure of body fat, such as DXA scans, and none has examined the spectrum of categories included in eNWO. The studies which investigated only the NWO category compared to a non-NWO category had similar results to the present study in that individuals with normal weight and high body fat had higher rates of metabolic dysfunction than those with normal weight and low body fat. For example, Madeira et al. investigated NWO and its relationship with insulin resistance and found that the presence of NWO, measured by skinfolds, was correlated with low insulin sensitivity compared to those of a normal weight without high body fat [6]. Additionally, Romero-Corral et al. studied the relationship between NWO and metabolic syndrome and found that NWO, when measured by bioelectrical impedance, was associated with four times the prevalence of metabolic syndrome compared to those in a normal weight-low body fat group [15]. Batsis et al. found that NWO, indexed using bioelectrical impedance, predicted higher insulin resistance when using tertiles, but not cut-points, for body fat [14]. In another investigation which used air displacement plethysmography to determine body fat percentage, findings showed that NWO predicted higher HOMA-IR levels [16]. Lastly, only one other study has used DXA to measure body fat, and this study found that NWO was associated with higher insulin resistance, but, like the others, it did not look at categories beyond normal weight-high body fat and normal weight-low body fat [17].

### 4.1. Potential Mechanisms

Currently, there is little known concerning the mechanisms associated with NWO as it relates to insulin resistance. However, there are a few potential mechanisms. First, plasma leptin concentrations are correlated with BMI and body fat levels [34]. Research shows that women with NWO have higher leptin levels than lean women, but lower levels than women with obesity [34]. Additionally, ghrelin, adiponectin, asprosin, and other hormones could be factors [35–37]. The amount of adipose tissue in the body significantly affects hormone levels [38], and hormones released by adipose tissue could contribute to insulin resistance [39]. Additionally, adipose cell size is predictive of metabolic dysregulation [40, 41]. Lastly, energy intake may be a moderating factor, affecting the relationship between insulin resistance and eNWO. Insulin resistance denotes a physiologic adaptation that may restrict the further storing of fat [42]. Some studies indicate that insulin resistance may protect against weight gain when body weight levels are extreme, but other studies show conflicting results [42].

### 4.2. Limitations and Strengths

The present study had multiple limitations. Due to the cross-sectional nature of the study, causality could not be determined. Moreover, the present study controlled several potential confounding variables, but there is always a possibility that an unknown lurking variable, not controlled in this investigation, was responsible for the relationship between eNWO and insulin resistance. Accuracy of self-reported variables was another potential limitation. Assessments of physical activity and smoking habits were both self-reported and, therefore, may contain errors due to misreporting. Lastly, insulin resistance was indexed using HOMA-IR. As noted by Yeni-Komshian et al. [43], there are better measures of insulin resistance.

A strength of the present study was its large sample size representing noninstitutionalized civilians in the United States. Because the sample represents virtually all of the United States adult population, the results are much more generalizable than the previous studies investigating NWO. Another strength was the use of DXA, a reliable and precise measure of body fat. Previous research has employed skinfolds, bioelectrical impedance, and other methods lacking the precision and reliability of DXA. Lastly, the present study expanded NWO, allowing the effect of body fat to be studied across each level of BMI. The concept of NWO has never been expanded before.

## 5. Conclusion

Both high BMI and high body fat percentage were strongly related to insulin resistance. However, according to the present study based on NHANES data, insulin resistance increased incrementally according to BMI levels primarily and body fat levels secondarily. Including a precise measure of body fat along with BMI seems to add little to the utility of BMI in the prediction of insulin resistance.

## Figures and Tables

**Table 1:** Expanded normal weight obesity (eNWO) categories, defined by BMI and body fat percentage status.

eNWO category	*N*, weighted %	Body mass index	Body fat % median^∗^
Underweight-low body fat	94, 1.9%	<18.5	Below
Underweight-high body fat	—	<18.5	Above
Normal weight-low body fat	1624, 30.1%	18.5–24.9	Below
Normal weight-high body fat	288, 4.5%	18.5–24.9	Above
Overweight-low body fat	927, 15.9%	25.0–29.9	Below
Overweight-high body fat	1221, 18.3%	25.0–29.9	Above
Obese-low body fat	141, 2.1%	≥30.0	Below
Obese-high body fat	1688, 27.2%	≥30.0	Above

^∗^Participants were divided according to body fat percentage based on the overall sex-specific median. Low body fat is defined as below the overall sex-specific median, and high body fat is defined as above the overall sex-specific median. Because the sample sizes for underweight-low and underweight-high were low, the two categories were merged to form one category, underweight. *N* represents the unweighted sample size, whereas weighted % represents the survey-weighted proportion of the total sample for each eNWO category. For weighted %, summing the values may not equal to 100% due to rounding. Primary focus should be on the proportions (%) because they represent the US population.

**Table 2:** Descriptive characteristics of the sample and mean HOMA-IR for each category (*n* = 5983).

Categorical variable	*N*	Weighted %	Mean HOMA	SE	*F*	*P*
eNWO					291.3	<0.0001
Underweight	94	1.9	0.8^a^	0.07		
Normal weight-low body fat	1624	30.1	1.1^b^	0.02		
Normal weight-high body fat	288	4.5	1.6^c^	0.08		
Overweight-low body fat	927	15.9	1.9^d^	0.05		
Overweight-high body fat	1221	18.3	2.2^e^	0.07		
Obese-low body fat	141	2.1	3.2^f^	0.28		
Obese-high body fat	1688	27.2	3.9^g^	0.14		
Sex					34.2	<0.0001
Men	2939	50.8	2.5^a^	0.09		
Women	3044	49.2	2.0^b^	0.05		
Race					15.0	<0.0001
Non-Hispanic White	2468	55.8	2.2^a^	0.09		
Non-Hispanic Black	847	7.9	2.5^b,d^	0.07		
Mexican American	1072	5.5	2.6^c,d^	0.08		
Other races	1401	26.8	2.3^a^	0.08		
Other Hispanics	195	3.9	2.4^d^	0.23		
Year of assessment					4.0	0.0114
1999–2000	1489	23.1	2.2^a,b^	0.08		
2001–2002	1679	26.9	2.1^b,c^	0.06		
2003–2004	1540	26.0	2.5^a^	0.16		
2005–2006	1275	23.9	2.3^c^	0.09		
Moderate physical activity					14.2	0.0004
Yes	3055	45.0	2.2^a^	0.07		
No	2928	55.0	2.4^b^	0.06		
Vigorous physical activity					30.1	<0.0001
Yes	2000	38.2	2.0^a^	0.06		
No	3983	61.8	2.4^b^	0.07		

NWO = expanded normal weight obesity category. Low body fat is defined as below the overall sex-specific median, and high body fat is defined as above the overall sex-specific median. The unweighted sample size (*N*) and the survey-weighted proportion (weighted %) of each subgroup are included. Focus should be on the survey-weighted proportions because they represent the US adult population. Summing the weighted % values may not equal to 100% due to rounding; The *F* values reflect mean HOMA-IR differences across each categorical variable without adjusting for any covariates. For each variable, means with the same superscript letters a, b, c, d, e, f, and g in the mean HOMA column are not significantly different (*P* > 0.05). Mean (±SE) age was 43.8 ± 0.4 years, and mean number of smoking pack-years was 0.8 ± 0.1. Among current smokers, the average number of cigarettes smoked per day was 17.9 ± 0.6.

**Table 3:** Mean differences in HOMA-IR across the expanded normal weight obesity (eNWO) categories, adjusted for covariates.

Covariates	Expanded normal weight obesity (eNWO)							*F*	*P*
	UW	NW-L	NW-H	OW-L	OW-H	OB-L	OB-H		
	mean ± SE	mean ± SE	mean ± SE	mean ± SE	mean ± SE	mean ± SE	mean ± SE		
None	0.8 ± 0.07^a^	1.1 ± 0.02^b^	1.6 ± 0.08^c^	1.9 ± 0.05^d^	2.2 ± 0.07^e^	3.2 ± 0.28^f^	3.9 ± 0.14^g^	291.3	<0.0001
Demographics^∗^	0.9 ± 0.09^a^	1.2 ± 0.05^b^	1.7 ± 0.08^c^	1.8 ± 0.07^d^	2.3 ± 0.08^e^	3.2 ± 0.28^f^	4.0 ± 0.13^g^	286.2	<0.0001
Demographics and lifestyle^†^	0.9 ± 0.09^a^	1.1 ± 0.05^b^	1.6 ± 0.09^c^	1.8 ± 0.07^d^	2.2 ± 0.09^e^	3.2 ± 0.29^f^	3.9 ± 0.12^g^	340.9	<0.0001

UW = underweight (*n* = 94, proportion = 1.9%); NW-L = normal weight-low body fat (*n* = 1624, proportion = 30.1%); NW-H = normal weight-high body fat (*n* = 288, proportion = 4.5%); OW-L = overweight-low body fat (*n* = 927, proportion = 15.9%); OW-H = overweight-high body fat (*n* = 1221, proportion = 18.3%); OB-L = obese-low body fat (*n* = 141, proportion = 2.1%); OB-H = obese-high body fat (*n* = 1688, proportion = 27.2%). *N*s are unweighted and proportions are survey-weighted. Focus should be on the survey-weighted proportions because they represent the US adult population. ^a,b,c,d,e,f,g^Means on the same row with the same superscript letter are not significantly different. For this table, each mean is significantly different from each other mean (*P* < 0.05). ^∗^Demographic covariates included age, sex, race, and year of assessment. ^†^Lifestyle covariates included moderate physical activity, vigorous physical activity, and smoking. Means on the same row have been adjusted for differences in the covariates listed in the first column.

**Table 4:** Mean HOMA-IR values across BMI sex-specific quintiles, without and with control of the covariates.

Variable controlled	BMI quintiles^§^					*F*	*P*
	Quintile 1 mean ± SE	Quintile 2 mean ± SE	Quintile 3 mean ± SE	Quintile 4 mean ± SE	Quintile 5 mean ± SE		
None	1.04 ± 0.03^a^	1.36 ± 0.03^b^	1.94 ± 0.05^c^	2.60 ± 0.08^d^	4.31 ± 0.17^e^	448.54	<0.0001
Demographics^∗^	1.10 ± 0.06^a^	1.41 ± 0.05^b^	1.99 ± 0.06^c^	2.65 ± 0.10^d^	4.37 ± 0.16^e^	431.10	<0.0001
Demographics and lifestyle^†^	1.11 ± 0.05^a^	1.42 ± 0.05^b^	2.01 ± 0.06^c^	2.65 ± 0.10^d^	4.35 ± 0.16^e^	464.39	<0.0001
Demographics, lifestyle, BF%5	1.48 ± 0.06^a^	1.59 ± 0.05^b^	2.02 ± 0.06^c^	2.50 ± 0.10^d^	3.95 ± 0.14^e^	141.88	<0.0001

^§^BMI quintiles are sex-specific. For each quintile, *n* represents the unweighted sample size and the percentage reflects the weighted proportion of the sample in that quintile, which represents the US adult population: Q1 (*n* = 1084; 20.0%), Q2 (*n* = 1135; 20.0%), Q3 (*n* = 1261; 20.0%), Q4 (*n* = 1262; 20.0%), Q5 (*n* = 1241; 20.0%). Total *n* = 5983. ^∗^Demographic covariates included age, sex, race, and year of assessment. ^†^Lifestyle covariates included moderate physical activity, vigorous physical activity, and smoking. Means on the same row have been adjusted for differences in the covariates listed in the first column. ^a,b,c,d,e^Means on the same row with the same superscript letter are not significantly different. For this table, each mean is significantly different from each other mean (*P* < 0.05). BF%5 represents the variable, body fat percentage, which was divided into sex-specific quintiles.

**Table 5:** Mean HOMA-IR values across body fat percentage quintiles, with and without control of the covariates.

Variable controlled	Body fat percentage quintiles^§^					*F*	*P*
	Quintile 1 mean ± SE	Quintile 2 mean ± SE	Quintile 3 Mean ± SE	Quintile 4 mean ± SE	Quintile 5 mean ± SE		
None	0.99 ± 0.03^a^	1.55 ± 0.5^b^	2.16 ± 0.07^c^	2.60 ± 0.07^d^	3.92 ± 0.17^e^	451.61	<0.0001
Demographics^∗^	0.98 ± 0.45^a^	1.59 ± 0.07^b^	2.24 ± 0.08^c^	2.72 ± 0.08^d^	4.06 ± 0.15^e^	448.21	<0.0001
Demographics and lifestyle^†^	1.01 ± 0.05^a^	1.60 ± 0.07^b^	2.25 ± 0.08^c^	2.71 ± 0.08^d^	4.04 ± 0.15^e^	511.33	<0.0001
Demographics, lifestyle, BMI5	1.80 ± 0.07^a^	2.10 ± 0.08^b^	2.38 ± 0.08^c^	2.34 ± 0.07^c^	2.91 ± 0.11^d^	68.83	<0.0001

^§^Body fat percentage quintiles are sex-specific. For each quintile, *n* represents the unweighted sample size and the percentage reflects the weighted proportion of the sample in that quintile, which represents the US adult population: Q1 (*n* = 1049; 19.8%), Q2 (*n* = 1137; 20.0%), Q3 (*n* = 1203; 19.9%), Q4 (*n* = 1283; 20.0%), and Q5 (*n* = 1311; 20.3%). Total *n* = 5983. ^∗^Demographic covariates included age, sex, race, and year of assessment. ^†^Lifestyle covariates included moderate physical activity, vigorous physical activity, and smoking. Means on the same row have been adjusted for differences in the covariates listed in the first column. BMI5 represents the variable, body mass index, divided into sex-specific quintiles. ^a,b,c,d,e^Means on the same row with the same superscript letter are not significantly different.

## References

[1] Poirier P., Giles T. D., Bray G. A. (2006). Obesity and cardiovascular disease: pathophysiology, evaluation, and effect of weight loss. *Arteriosclerosis, Thrombosis, and Vascular Biology*.

[2] Flegal K. M., Carroll M. D., Kuczmarski R. J., Johnson C. L. (1998). Overweight and obesity in the United States: prevalence and trends, 1960-1994. *International Journal of Obesity and Related Metabolic Disorders: Journal of the International Association for the Study of Obesity*.

[3] Ogden C. L., Carroll M. D., Kit B. K., Flegal K. M. (2014). Prevalence of childhood and adult obesity in the United States, 2011-2012. *Journal of the American Medical Association*.

[4] Lim S. M., Choi D. P., Rhee Y., Kim H. C. (2015). Association between obesity indices and insulin resistance among healthy Korean adolescents: the JS high school study. *PLoS One*.

[5] Hillier T. A., Pedula K. L. (2001). Characteristics of an adult population with newly diagnosed type 2 diabetes: the relation of obesity and age of onset. *Diabetes Care*.

[6] Madeira F. B., Silva A. A., Veloso H. F. (2013). Normal weight obesity is associated with metabolic syndrome and insulin resistance in young adults from a middle-income country. *PLoS One*.

[7] De Lorenzo A., Bianchi A., Maroni P. (2013). Adiposity rather than BMI determines metabolic risk. *International Journal of Cardiology*.

[8] Gallagher D., Heymsfield S. B., Heo M., Jebb S. A., Murgatroyd P. R., Sakamoto Y. (2000). Healthy percentage body fat ranges: an approach for developing guidelines based on body mass index. *The American Journal of Clinical Nutrition*.

[9] Okorodudu D. O., Jumean M. F., Montori V. M. (2010). Diagnostic performance of body mass index to identify obesity as defined by body adiposity: a systematic review and meta-analysis. *International Journal of Obesity (London)*.

[10] Romero-Corral A., Somers V. K., Sierra-Johnson J. (2008). Accuracy of body mass index in diagnosing obesity in the adult general population. *International Journal of Obesity (London)*.

[11] Baumgartner R. N., Heymsfield S. B., Roche A. F. (1995). Human body composition and the epidemiology of chronic disease. *Obesity Research*.

[12] Oliveros E., Somers V. K., Sochor O., Goel K., Lopez-Jimenez F. (2014). The concept of normal weight obesity. *Progress in Cardiovascular Diseases*.

[13] Batsis J. A., Sahakyan K. R., Rodriguez-Escudero J. P., Bartels S. J., Lopez-Jimenez F. (2014). Normal weight obesity and functional outcomes in older adults. *European Journal of Internal Medicine*.

[14] Batsis J. A., Sahakyan K. R., Rodriguez-Escudero J. P., Bartels S. J., Somers V. K., Lopez-Jimenez F. (2013). Normal weight obesity and mortality in United States subjects >/=60 years of age (from the third National Health and Nutrition Examination Survey). *The American Journal of Cardiology*.

[15] Romero-Corral A., Somers V. K., Sierra-Johnson J. (2010). Normal weight obesity: a risk factor for cardiometabolic dysregulation and cardiovascular mortality. *European Heart Journal*.

[16] Gomez-Ambrosi J., Silva C., Galofre J. C. (2012). Body mass index classification misses subjects with increased cardiometabolic risk factors related to elevated adiposity. *International Journal of Obesity (London)*.

[17] Kim M. K., Han K., Kwon H.-S. (2014). Normal weight obesity in Korean adults. *Clinical Endocrinology*.

[18] Cauley J. A. (2015). An overview of sarcopenic obesity. *Journal of Clinical Densitometry: The Official Journal of the International Society for Clinical Densitometry*.

[19] Lu C. W., Yang K. C., Chang H. H., Lee L. T., Chen C. Y., Huang K. C. (2013). Sarcopenic obesity is closely associated with metabolic syndrome. *Obesity Research & Clinical Practice*.

[20] Chung J. Y., Kang H. T., Lee D. C., Lee H. R., Lee Y. J. (2013). Body composition and its association with cardiometabolic risk factors in the elderly: a focus on sarcopenic obesity. *Archives of Gerontology and Geriatrics*.

[21] Kim T. N., Park M. S., Lim K. I. (2013). Relationships between sarcopenic obesity and insulin resistance, inflammation, and vitamin D status: the Korean sarcopenic obesity study. *Clinical Endocrinology*.

[22] NHANES About the National Health and Nutrition Examination Survey: National Center for Health Statistics; 1960–2017. https://www.cdc.gov/nchs/nhanes/about_nhanes.htm.

[23] NHANES Data files: questionnaires, datasets, and related documentation: Centers for Disease Control and Prevention; 1999–2002. http://www.cdc.gov/nchs/nhanes/nhanes_questionnaires.htm.

[24] NHANES The 1999–2006 dual energy X-ray absorptiometry (DXA) multiple imputation data files and technical documentation Hyattsville, MD: U.S. Department of Health and Human Services, Centers for Disease Control and Prevention; 1999–2006. http://www.cdc.gov/nchs/nhanes/dxx/dxa.htm.

[25] NHANES (2000). *National Health and Nutrition Examination Survey Examination Protocol*.

[26] ACSM (2014). *ACSM's Guidelines for Exercise Testing and Prescription*.

[27] Schenker N., Borrud L. G., Burt V. L. (2011). Multiple imputation of missing dual-energy X-ray absorptiometry data in the National Health and Nutrition Examination Survey. *Statistics in Medicine*.

[28] NHANES National Health and Nutrition Examination Survey Laboratory Protocol Hyattsville, MD: National Center for Health Statistics; U.S. Department of Health and Human Services; 1999–2006. https://www.cdc.gov/nchs/nhanes/nhanes_questionnaires.htm.

[29] NHANES National Health and Nutrition Examination Survey Questionnaire, Hyattsville, MD: U.S. Department of Health and Human Services, Centers for Disease Control and Prevention; 1999-2000. http://www.cdc.gov/nchs/nhanes/nhanes1999-2000/questionnaires99_00.htm.

[30] Needham B. L., Adler N., Gregorich S. (2013). Socioeconomic status, health behavior, and leukocyte telomere length in the National Health and Nutrition Examination Survey, 1999-2002. *Social Science & Medicine*.

[31] Wei Y., Zhu J., Nguyen A. (2014). Urinary concentrations of dichlorophenol pesticides and obesity among adult participants in the U.S. National Health and Nutrition Examination Survey (NHANES) 2005-2008. *International Journal of Hygiene and Environmental Health*.

[32] SAS SAS 9.4 documentation by title. http://support.sas.com/documentation/cdl_main/94/docindex.html.

[33] Gomez-Ambrosi J., Silva C., Galofre J. C. (2011). Body adiposity and type 2 diabetes: increased risk with a high body fat percentage even having a normal BMI. *Obesity (Silver Spring, Maryland)*.

[34] Marques-Vidal P., Pecoud A., Hayoz D. (2010). Normal weight obesity: relationship with lipids, glycaemic status, liver enzymes and inflammation. *Nutrition, Metabolism, and Cardiovascular Diseases: NMCD*.

[35] Romere C., Duerrschmid C., Bournat J. (2016). Asprosin, a fasting-induced glucogenic protein hormone. *Cell*.

[36] Huang W. Y., Chang C. C., Chen D. R., Kor C. T., Chen T. Y., Wu H. M. (2017). Circulating leptin and adiponectin are associated with insulin resistance in healthy postmenopausal women with hot flashes. *PLoS One*.

[37] Al Qarni A. A., Joatar F. E., Das N. (2017). Association of plasma ghrelin levels with insulin resistance in type 2 diabetes mellitus among Saudi subjects. *Endocrinology and Metabolism*.

[38] Nway N. C., Sitticharoon C., Chatree S., Maikaew P. (2016). Correlations between the expression of the insulin sensitizing hormones, adiponectin, visfatin, and omentin, and the appetite regulatory hormone, neuropeptide Y and its receptors in subcutaneous and visceral adipose tissues. *Obesity Research & Clinical Practice*.

[39] You T., Wang X., Murphy K. M. (2014). Regional differences in adipose tissue hormone/cytokine production before and after weight loss in abdominally obese women. *Obesity (Silver Spring, Maryland)*.

[40] McLaughlin T., Craig C., Liu L. F. (2016). Adipose cell size and regional fat deposition as predictors of metabolic response to overfeeding in insulin-resistant and insulin-sensitive humans. *Diabetes*.

[41] Eriksson-Hogling D., Andersson D. P., Backdahl J. (2015). Adipose tissue morphology predicts improved insulin sensitivity following moderate or pronounced weight loss. *International Journal of Obesity (London)*.

[42] Tucker L. A., Tucker J. M. (2012). Insulin resistance as a predictor of gains in body fat, weight, and abdominal fat in nondiabetic women: a prospective study. *Obesity*.

[43] Yeni-Komshian H., Carantoni M., Abbasi F., Reaven G. M. (2000). Relationship between several surrogate estimates of insulin resistance and quantification of insulin-mediated glucose disposal in 490 healthy nondiabetic volunteers. *Diabetes Care*.

